# Alternative Splicing by NOVA Factors: From Gene Expression to Cell Physiology and Pathology

**DOI:** 10.3390/ijms21113941

**Published:** 2020-05-30

**Authors:** Jacopo Meldolesi

**Affiliations:** Department of Neuroscience, San Raffaele Institute and San Raffaele University, via Olgettina 58, 20132 Milan, Italy; meldolesi.jacopo@hsr.it

**Keywords:** GTEx: a Portal Project Consortium, L1CAM: a target protein of NOVA2, NOVA1 and NOVA2: splicing factors of the NOVA family, pre-mRNA: a mRNA before its alternative splicing, REST: a negative transcription factor, spliceosome: a complex of an alternative splicing factor

## Abstract

NOVA1 and NOVA2, the two members of the NOVA family of alternative splicing factors, bind YCAY clusters of pre-mRNAs and assemble spliceosomes to induce the maintenance/removal of introns and exons, thus governing the development of mRNAs. Members of other splicing families operate analogously. Activity of NOVAs accounts for up to 700 alternative splicing events per cell, taking place both in the nucleus (co-transcription of mRNAs) and in the cytoplasm. Brain neurons express high levels of NOVAs, with NOVA1 predominant in cerebellum and spinal cord, NOVA2 in the cortex. Among brain physiological processes NOVAs play critical roles in axon pathfinding and spreading, structure and function of synapses, as well as the regulation of surface receptors and voltage-gated channels. In pathology, NOVAs contribute to neurodegenerative diseases and epilepsy. In vessel endothelial cells, NOVA2 is essential for angiogenesis, while in adipocytes, NOVA1 contributes to regulation of thermogenesis and obesity. In many cancers NOVA1 and also NOVA2, by interacting with specific miRNAs and by additional mechanisms, activate oncogenic roles promoting cell proliferation, colony formation, migration, and invasion. In conclusion, NOVAs regulate cell functions of physiological and pathological nature. Single cell identification and distinction, and new therapies addressed to NOVA targets might be developed in the near future.

## 1. Introduction

For many decades, gene expression has often been identified as the process of DNA gene transcription by the specific polymerase II enzyme into the corresponding mRNAs. The mRNAs are then believed to start a series of events ultimately leading to the biogenesis of the specific proteins. In general terms, such a classical definition of gene expression is valid. However, intense studies carried out during the last three decades have demonstrated that gene-RNA conversion is more complex than previously believed. In most cases, in fact, the RNAs matching the corresponding DNA sequences, now defined as pre-mRNAs, undergo a removal of internal sequences called introns, which can vary in number and position along the transcript body. The fractions with a lower chance of being removed, called exons, account for the conversion of pre-mRNAs into mRNAs. The latter process is due to a number of parallel active processes, the alterative splicing processes.

Alternative splicing is among the most important regulatory processes existing in the cells. From 70% to 90% of the neo-synthesized pre-mRNAs undergo splicing processes, variable from two to a thousand per gene. The average number of distinct splicing processes occurring per cell has been calculated to be around 10,000. Each pre-mRNA can account for two to about 1000 splicing events, accounting for the apparent diversity of its nature and for the multiplicity of its protein products that can be diverse in terms of composition, structure and function [1,2]. In all cells, the number of mRNAs is therefore larger, in many cells much larger, than the number of genes transcribed from the DNA. Another difference among splicing events depends on their location. Numerous, and in many cases predominant events, take place in the course of gene transcription, i.e., co-transcriptionally. Other events, however, take place post-transcriptionally, coupled with subsequent processes such as RNA export from the nucleus and various cytoplasmic processes [1,2,3,4,5].

The splicing factors do not operate alone, but together with numerous other proteins and RNAs combined into large complexes called spliceosomes, which are assembled once for each splicing event, sustained by the dynamic interactions among their RNA and protein components. In the co-transcriptional splicing events, the spliceosomes do not operate independently but by integration and reciprocal modulation with the transcription machineries [6].

As already mentioned, alternative splicing is present in almost all types of cells. Such activity depends on the expression of specific factors, many of which are abundant in the brain. Among these factors are the Neuro-Oncological Ventral Antigens 1 and 2, usually called by their abbreviations NOVA1 and NOVA2, defined for many years as neuron-specific. Of the numerous splicing factors, the NOVAs, two similar proteins from a single family that governs the alternative splicing of a variety of pre-mRNAs, are profoundly known and understood. By such a process, large numbers of introns, and in some cases also exons, are removed from pre-mRNAs, with the generation of mRNAs active in stem, growing, and mature cells. As a consequence, single genes ultimately encode multiple protein isoforms, often with distinct structural and functional properties [7,8].

NOVAs and their splicing properties were discovered over three decades ago. Since then, the mechanisms and functions of the two splicing factors have been extensively investigated, with growing interest in the physiological and pathological processes in which NOVAs are involved. The present review deals with these processes dependent on the two NOVAs. Most such processes occur co-transcriptionally, i.e., in the course of gene expression. Thus, the participation of this review in a Special Issue dealing with gene expression is appropriate. By analyzing the present knowledge about NOVAs I realized that in numerous previous studies, published in excellent journals from 2000 to 2015, the extensive properties of the factors were those active in brain neurons. A complete presentation of these findings can be found in previous reviews of the field [9,10]. During the last five years, these studies have been extended, with the identification of new and critical properties of neurons. In the meantime, NOVA studies have been extended to the physiology and pathology of non-neuronal cells, including many cancers, and to the first perspectives of therapy. Based on these considerations, I have decided to focus this review on a correlation between a fraction of classical knowledge, established during the last decades, and innovative developments discovered during the last five years.

## 2. Gene Expression and Alternative Splicing of NOVAs

In this Section, I report about two general aspects of NOVA generation and function concerning (1) the expression of NOVA genes in neurons and non-neuron cells and (2) the differential properties of their alternative splicing. To answer the first question, I have investigated in NOVAs the possible role of REST, a negative transcription factor, the most important regulator of neural-type gene expression. To investigate the second question, I have reconsidered many properties of NOVAs of relevance for their physiological and pathological properties of the cells.

### 2.1. Mechanisms of NOVA Gene Expression

My first aspect, so far little investigated, concerns the expression mechanisms of the two alternative splicing factor genes in neurons and non-neural cells. For many other genes, the differential expression between these two types of cells depends on REST, a well-known negative transcription factor. In all types of stem cells, the REST gene is highly expressed. However, during cell maturation, the levels of REST become different: it remains high in non-neuronal cells, whereas it declines to almost inappreciable levels in neurons and neuron-like cells. In stem cells and mature non-neuronal cells, the high level of REST prevents the expression of many neuron-specific genes, while in mature neurons, the very low levels of REST makes possible the expression of such genes [11,12]. The relevance of the REST level has been confirmed by studies in PC12, a well-known neuronal-like cell line. In most clones isolated from these cells, the level of REST is as low as in mature neurons, while in a few other clones, the level of REST is high as in non-neural cells. The comparative study of the low and high REST clones revealed that, of the almost 14,000 genes expressed in this cell line, about 900 were more than two-fold higher and about 900 more than two-fold lower in the high versus the low REST clones. In PC12 cells, therefore, the expression of about 1800 genes appears governed by REST [13].

Are the genes of the two NOVAs expressed under the control of REST? In studies carried out in PC12 and also in human neuron-like cell lines (SH-SY5Y, NT2/D1) and rat mature neurons, NOVA1 gene expression was the same at low and high levels of REST. In contrast, NOVA2 gene expression was about five-fold higher in the low REST, typical of neurons and neuron-like cells, than in the high REST clones [13,14]. Such REST dependence of NOVA2 expression was confirmed by results in one of its targets, the well-known adhesion/signaling protein L1CAM. In the PC12 clones, L1CAM exhibits expected properties. In the wild-type PC12 clones, characterized by low REST, the mRNA sequence of L1CAM was complete, and in the high REST clones, it was spliced [14]. I conclude that REST is important for the expression of the NOVA2 gene, and not for the NOVA1 gene. This does not exclude that other transcription factors, working together or independently of REST, are involved in the expression of NOVA-dependent genes. The different mechanisms of expression of the two NOVA genes in the neurons of various brain areas (and possibly also in different non-neuronal cells) could be due, at least in part, to their different dependence on REST.

### 2.2. Alternative Splicing of the Two NOVAs

**Figure 1:**
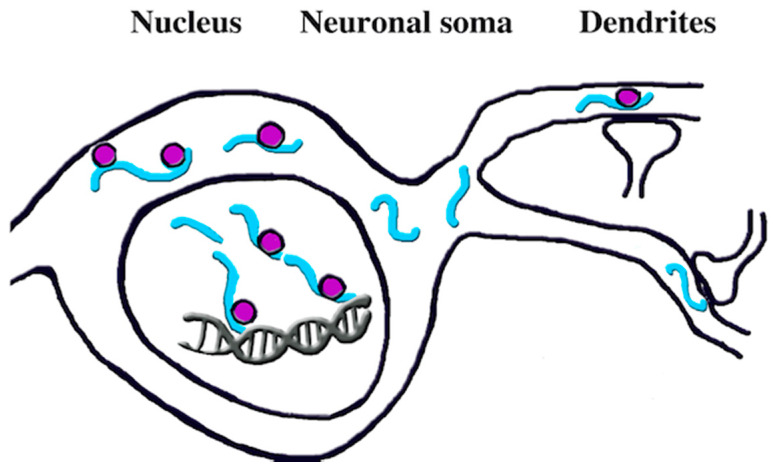
Pre-mRNAs-mRNAs conversion induced by NOVAs. The splicing occurs predominantly in the nucleus where spliceosome complexes (red spheres) bind to YCAY motifs and remove introns from growing pre-mRNAs (co-transcriptional process). Further splicing takes place in the cytoplasm (including dendrites near synapses), with spliceosomes operative on pre-mRNAs (pre-translation process), making possible their subsequent translation. The long blue filaments are pre-mRNAs, the shorter filaments are the results of ongoing or complete conversion into mRNAs. (details from Ref. [24]).

As already mentioned in Section 1, alternative splicing occurs by multi-component machine complexes, the splicesomes, composed by various proteins and RNAs. During the process, the complexes undergo dynamic arrangements via RNA–RNA, protein–protein, and RNA–protein interactions. Co-transcriptional splicing, the most frequent process of NOVAs, is based on the functional integration of the splicing and transcriptional machineries [6]. Many of the effects induced by NOVAs in their conversion of pre-mRNAs to the final mRNAs take place by alternative splicing events occurring in the course of transcription of the specific genes (Figure 1). The generation of mRNAs by such alternative splicing and the biogenesis of the corresponding proteome targets take place sequentially. These splicing events therefore contribute to two relevant protein results: the multiplicity of the protein forms encoded by a single gene and the specificity of their level and function in distinct cells and tissues [7,8,9,10].

Alternative splicing is not the only process regulated by the NOVA-RNA targets. For example, in CA1 pyramidal neurons, NOVA2 is necessary for long-term potentiation responses mediated by the GIRK2 kinase channel and GABA(B) receptor [15]. However, mechanistic details of such NOVA effect remain unclear. Another NOVA process that attracts growing interest is alternative polyadenylation, a post-transcriptional process by which various mRNAs with different 3′UTRs are generated from single genes [16]. Polyadenylation, now investigated in single brain cells by an advanced approach [17], is largely modulated by NOVA1 and NOVA2 [18]. Polyadenylation, however, is distinct from alternative splicing. Therefore, it is not further mentioned in the present review.

Knowledge of alternative splicing has been significantly increased by the combination of various computational and experimental approaches. Intense studies have identified several effects induced by NOVA1 and NOVA2 working apparently together. However, the reciprocity of the two factors is not general. This explains how, in cells expressing both, some splicing effects depend on one factor and not on the other. Specific binding of NOVAs to their pre-mRNA targets occurs at clusters of a four-nucleotide motif (pyrimidine, cytosine, adenine, pyrimidine: YCAY), distributed in the proximity of introns and exons [19]. Depending on the variable distribution of the clusters, the action of NOVAs can result in the inclusion or release of introns, in some cases also of exons. Accurate mapping of RNAs has made possible the prediction of the NOVA-dependent regulation of splicing. In vivo, the two NOVA factors regulate independently at least 700 alternative splicing events [19]. Many other events are governed by splicing factors belonging to different families.

Upon their discovery, NOVA1 and NOVA2 were shown to operate during both neuronal development and maturity. Various studies revealed their distribution among brain areas to be largely distinct, with NOVA1 predominant in the ventral spinal cord, and NOVA2 predominant in the cortex. Neurons, however, are not the only cell targets of NOVAs. Some level of NOVA2 in adult lung cells was first reported in an early study [20]. Relevance of NOVA1 was noticed and then confirmed in various types of cancers [21]. Recently, the differential expression of the two NOVA genes has been established in human cell populations by the analyses of the GTEx Portal Project Consortium [22,23]. In the brain, the average gene expression of NOVA1 has been found about three-fold higher than that of NOVA2. In the cerebellum, the predominance of NOVA1 gene expression is high, while in the spinal cord, hippocampus, and substantia nigra, such predominance is lower. In the other areas of the brain the difference is minor (Table 1). Outside the brain, NOVA gene expression is lower than in the brain. Predominance of NOVA1 is in adipose, mammary tissue, cervix, colon, and muscle cells, and that of NOVA2 in the lung, where NOVA1 is almost lacking (Table 1). In many other cells, the gene expression of both NOVAs is very low or absent [22,23].

## 3. Levels and Functions of NOVAs in Neurons and Other Cells

As previously mentioned, the definition of NOVAs as neuron-specific, started upon their discovery, has been employed for several years. Over 20 years ago, however, NOVA2 had been reported to exist also in the lung [20]. Within subsequent years, the extra-neuronal distribution of NOVA mRNAs and proteins, however at levels lower than those in neurons, was reported in many other tissues and a variety of cancers [21,22,23]. The properties of NOVAs in neurons, neurosecretory, and non-neural cells are reported in the following Section 3.1, Section 3.2 and Section 3.3.

### 3.1. Neurons

Extensive studies have revealed numerous and important properties of NOVAs expressed in neurons. As in other cells, the neuronal splicing factors operate within the intracellular organ of gene transcription, the nucleus. However, in neurons, the NOVAs have been shown to shuttle between the nucleus and the cytoplasm, with about 50% of the protein localized in both such compartments (Figure 1). It appears, therefore, that the intracellular localization of the two factors depends on the regulation between nuclear generation and intracellular RNA distribution [24,25]. In both compartments, however, the level and distribution of the two processes are not stable but can change depending on various stimuli.

In the cytosolic compartment of neurons, NOVA activities are often of great importance. During development, deficiencies of NOVA2 ultimately result in defects of axon pathfinding and spreading in the brain, with ensuing agenesis of corpus callosum accompanied by defects of motoneuron axons and cochlear innervation. Alterations of axons also induce defects of neuron migration [25,26]. Dendrites are affected, especially in the areas beneath synapses, where the NOVAs bind mRNAs participating in the synthesis of localized proteins [25,26,27] (Figure 1). Neocortex analyses of spliced RNAs has led to the identification of various NOVA2-dependent protein forms necessary for the establishment, structure and function of synapses [28]. Some of the additional processes regulated by splicing factors are located at the cell surface, dependent on receptors and channels. Interestingly, splicing by NOVA2 is different in excitatory and inhibitory neurons. Such differences are relevant in both cortical development and cerebellar function [29]. Summing up, NOVAs orchestrate changes in the development, structure, and function of neurons including their synapses.

Receptors regulated by NOVAs are of various types. Ample evidence is present about subunits. The latter include the γ-subunit of GABA(A) and glycine receptors, of great relevance at inhibitory synapses [30,31], as well as the nicotinic receptor, a stimulatory surface channel relevant at neuromuscular junctions and many brain synapses [32,33]. In addition, NOVA regulation occurs at G protein receptors. For example, NOVA1 has been recently found to regulate the serotonin receptor 5HT_6_, not only via specific mRNAs but apparently also by direct binding [34]. In addition to receptors, NOVA regulation has been demonstrated for voltage-gated channels. Two members of the N and P Ca^2+^ channel families are similarly spliced by NOVA2, with analogous effects induced by possible removal of two exons [35]. The Na^+^ voltage-gated channels are critical, not only in mammals but also in lower animals, down to fruitflys. In this case, regulation is by NOVA2 splicing, operative also on variants of the SCN1A channel [36,37].

### 3.2. Neurosecretory Cells

Although different in many respects, these cells are shown to share many functional properties with neurons. Therefore, their NOVA expression was not unexpected. I have already presented rat PC12 [13,14], a line derived from a cancer of adrenal medulla cell origin, heterogeneous in its numerous isolated clones. In PC12 cells high expression of NOVA2 was found in clones competent for neurosecretion and much lower in clones incompetent for that function [13]. Extensive studies have been carried out also in another type of neurosecretory cells, the rat and human β-cells of the pancreas. In these cells NOVA1, participating in the splicing of numerous pre-mRNAs, is needed for the synthesis and release of insulin [38]. Expression of NOVA2 in these cells was demonstrated in a subsequent study. Together with another splicing factor, Elavl4, NOVA2 was found to protect β-cells from apoptosis [39]. Taken together, the results obtained so far confirmed that neurosecretory cells share with neurons both regulation and mechanistic programs of NOVA expression and function.

### 3.3. Non-Neural Cells

The first non-neural cells regulated by alternative splicing was observed in 1998 in the lung [20], an organ where non-neural expression of NOVA2 is significant [23] (Table 1). Recently, the relevance of the two NOVA factors has been demonstrated in other cells distinct from neurons, which have attracted great attention. Two examples of these cells are illustrated in this Subsection.

#### 3.3.1. Endothelial Cells

**Figure 2:**
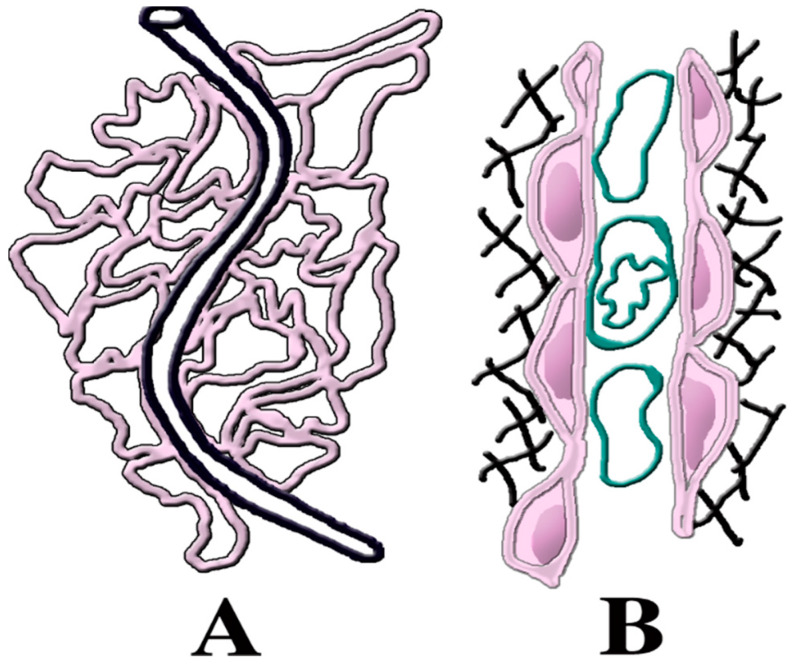
NOVA2 is needed for angiogenesis. (**A**) A low magnification drawing showing angiogenesis in a network of small vessels positive for NOVA2 (rose) growing from a pre-existing vessel (black). (**B**) A growing vessel is shown at higher magnification. The NOVA2-positive flat cells (rose) are endothelia adjacent to the lumen. The latter contains uncolored images of blood cells. The networks on both sides correspond to structures adjacent to the external face of endothelial cells.

In recent years, the NOVA-dependent process that has received great attention is angiogenesis, i.e., the process by which vessels are generated, especially during the development and restoration of organs and in growing cancers. In angiogenesis the coordinate process is vascular lumen formation, sustained by proliferation of endothelial cells. Direct studies demonstrated that, without NOVA2, the polarity of endothelial cells, required for vascular lumen formation, is altered, thus growing vessels are disrupted. In other words, NOVA2 expression by endothelial cells (Figure 2) is necessary for angiogenesis to occur [40].

The mechanism of this effect has been investigated recently. In endothelial cells, alternative splicing by NOVA2 was shown to results in the generation of several proteins encoded by genes such as Ppar-γ and E2F1. At least some of these spliced proteins appear critical for the endothelial cell function [41]. Additional studies by the same group have recognized the role of NOVA2 in the splicing of a surface adhesion protein, L1CAM. Upon the loss of the two introns already mentioned [14], the spliced isoform of the protein is released from the plasma membrane. Together with the signaling of an FGF receptor, spliced L1CAM contributes towards the progress of angiogenesis. Such mechanistic process occurs also for vessel generation in the ovarian cancer [40]. Thus, its relevance is wide, important for general vein growth and also for future developments of cancer therapy [42]. Interestingly, a possibility analogous to the latter emerged also from NOVA2 studies about the vasculature of colorectal cancers [43]. The role of NOVA2 about the endothelial cells of veins, discussed so far, has been shown to operate also in the corresponding cells of lymphatic vessels. In this case, the splicing factor operates on the activity of phosphokinases such as Erk [44]. In vessels, therefore, NOVA2 operates in the assignment and the polarity of endothelial cells, acquired during vascular growth and differentiation (Figure 2).

#### 3.3.2. Adipocytes

Non-neuronal cells of another type, affected by NOVAs and other splicing factors, are adipocytes. NOVA1 has been shown to govern thermogenesis and also glycemia, important for the establishment and the control of obesity [45]. Another type of adipocyte is converted into the brown form by a metabolic cascade, including an RNA-binding protein spliced by NOVA1. In addition, NOVA1 interacts with and affects other proteins of the cell. Although different during cell development and upon maturation, the NOVA1 cascade maintains an important role, essential for the structure and function of adipocytes [46].

## 4. Diseases and Therapies

NOVA splicing factors are relevant not only in the physiology of neurons and some non-neuronal cells, but also in the corresponding diseases, including neurologic diseases and a large number of cancers [21,47]. Knowledge about the properties of NOVAs action can ultimately contribute to the understanding of the various types of diseases. In these cases, NOVAs operate in distinct processes, including excitation of neurons and cell proliferation and migration in cancers. At present, new types of therapy, dependent on NOVAs mechanisms of action, have been envisaged, but not yet practically developed. In other cases, therapy has not been even considered. Based on the above considerations, the present Section has been structured in three Subsections dealing with: (1) neurologic and non-neurologic diseases, (2) cancers, and (3) perspective therapies.

### 4.1. Neurologic and Non-Neurologic Diseases

The importance of alternative splicing in the pathogenesis of neuronal disturbances and diseases has already been mentioned. Here, the problem is considered in more detail [48,49,50,51,52,53,54,55,56,57,58,59]. In a list of diseases, including, for example, ataxia-telangectasia, various muscle dystrophies, and spinal muscle atrophies, defects appear to be sustained by splicing factors not yet identified [49]. A role of NOVA2 in patients affected by the rare POMA disease had been demonstrated many years ago [20]. Other diseases have been suggested years ago, but never demonstrated.

Possible roles of NOVAs have been revealed by recent findings. The contribution of the RNA-binding protein TDP-43, operative together with NOVA1, may contribute to mechanistic lesions in amyotrophic lateral sclerosis, fronto-temporal dementia, various neurodegenerative diseases and schizophrenia [50]. Likewise, cooperative NOVA1 and NOVA2, working together with miRNAs, have been hypothesized to participate in the pathogenesis of intellectual disabilities, familial dysautonomia, and bipolar disorders [51,52,53]. The RNA-binding protein RBM8A, spliced by various factors including NOVA1, induce defects relevant also in Alzheimer’s disease [54]. In such disease NOVAs appear to contribute in changes of RNA metabolism [55]. Some forms of another important disease, epilepsy, is regulated by NOVA2 [36,56]. In particular, the splicing factor interacts with voltage-gated Na^+^ channels relevant in the activation of brain mechanistic lesions [36,56]. Within diseases of non-neural cells, NOVAs defects have been shown to contribute in anti-angiogenesis [42], obesity and alterations of glycemia [45].

### 4.2. Cancers

As already mentioned [21], the main extra-neuronal effects of NOVAs concern cancers, where the alternative splicing factors often promote cell proliferation, colony formation, migration and invasion. Interestingly, the cancers regulated by NOVA1 include not only those of the brain, but also many others. NOVA2 operates less frequently, but is not absent.

The mechanism by which NOVA1 induces stimulation of cancer cells often includes its interaction to miRNA. Interestingly, the miRNAs involved in various cancers is often different. In glioblastoma, the reported miRNA is miR-193a-5p [58], in astrocytoma, miR-181b-5p [59], in thyroid cancer, miR-592 [60], in breast cancer, miR-140-3p [61], in gastric cancer, miR-339 [62]; in the disease cells residual after gastric cancer removal, miR-146b [63], in myeloma, miR-181a [64], and in osteosarcoma, miR-146a [65]. In all these cancer cells, the effects of NOVA1 is oncogenic, operative via the binding and inhibition of the miRNAs mentioned above, which have a cell protective role [58,59,60,61,62,63,64,65].

The oncogenic effect of NOVA1 via inhibition of miRNAs is not a rule. In a colorectal cancer NOVA1 has a stimulatory role, active however not against but in cooperation with the mRNA of interleukin 6, with ensuing stimulation of JAK2 and STAT3 signaling [66]. Examples exist in which the alternative splicing factor plays a protective role. This is the case of a gastric cancer expressing another miRNA, miR-27a-3p, which operates as an oncogene and promotes an epithelial-mesenchymal transition, whereas NOVA1 prevents cell proliferation [67]. The slow growth of another gastric cancer depends primarily on microenvironment, with predominance of immune cells. In the latter cells high NOVA1 is protective whereas a low level is associated with tumor progression and poor prognosis [68]. MiRNA dependence has been shown also for NOVA2. In breast cancer, NOVA2 operates together with NOVA1 to increase the stability of β-catenin, a protein that increases the epithelial-mesenchymal transition [69]. In other cases, NOVA2 operates alone. miR-7-5p has been shown to inhibit in vivo and in vitro two types of cancers, the non-small cell lung cancer and the human glioma. In the first NOVA2 attenuates the inhibitory effect of miR-7-5p, in the second the effect of NOVA2 is not inhibited but shared by another RNA, a non-coding form known to operate in a miRNA sponge [70,71].

The interaction of NOVAs with miRNAs is the most frequent, but not the only mechanism governing their effects on cancers. Among additional mechanisms regulated by NOVA1 is the increased telomerase activity, with ensuing telomere maintenance together with increased cancer cell proliferation [72,73]. In melanoma, NOVA1 inhibits the protein FOXO3A and thus stimulates proliferation [74]. Another mechanism active in vivo is the co-operation of NOVA1 with the GABA(A) receptor 2, which operates as oncogene in various tissues [75]. Stimulation of ovarian and colorectal cancers is induced also by NOVA2 working indirectly, via stimulation of tumor angiogenesis [42,43].

Summing up, the effect in cancer induced by NOVA1 and NOVA2 is most often cell proliferation. In other cases, however, the effect is down-regulation dependent on various factors, including specific miRNAs and the environment. The variability of these effects is of potential interest not only for the diagnosis, but also for the generation and use of valuable prognostic biomarkers in individual cancer patients.

### 4.3. Potential Therapy

The multiple effects of NOVA factors, including their function analogous to other splicing factors, the organization of their spliceosomes and the multiplicity of their targets, are often considered of potential interest for the development of innovative therapies. Up to now, however, effective therapies have not been reported. Here, therefore, the presentation is limited to the few articles in which therapies have been mentioned, of potential interest for future developments.

An opportunity can be hypothesized based on a well-known pharmacological approach applied to SCN1A, a voltage-gated channel so far of limited importance in pharmacology [37]. Additional approaches are recommended to treat various neurological and psychological diseases. The expected results are the identification of potential co-biomarkers, with ensuing treatments for stress-induced neuropsychiatric disorders [48,50,52]. In other papers, attempts starting from a splicing factor are hypothesized to play a role in the development of innovative drugs active in some forms of epilepsy and forms of stress [36,48,52,76]. Additional attempts for the development of new therapies have been raised also for various brain and lung cancers [70,71,75]. Finally, new therapies against anti-angiogenic cancers have been proposed based on the control of L1CAM expression by NOVA2 splicing [41,42].

## 5. Conclusions

Starting from their general properties, alternative splicing processes are known to operate from gene transcription to gene product multiplication. In these fields interest has been focused on NOVA, a family of splicing factors well known for its cellular effects. Knowledge about neuronal physiology, accumulated during the last two decades, has been strengthened recently, especially by results about axons and synapses, and expanded by results about two non-neuronal processes. Concomitantly, studies have been established to investigate pathology, neurodegeneration, and other neuronal and non-neuronal diseases. Much more developed have been those about cancer, where a predominant role of NOVA1 has been demonstrated. It can be concluded, therefore, that present interest about NOVAs is largely focused on both physiology and pathology, although knowledge about the various aspects is variable: considerable in some, only limited in others.

For the future, the development that can be expected is still wide. General knowledge about the various brain areas is already well established. In some cases, however, the possible distinction among cell types existing in single brain areas, concerning also the various non-neural cells, remains to be investigated. In other non-neural organs and tissues, such as lung, mammary gland, uterus cervix, colon, and muscles, expressing considerable levels of NOVAs, no studies have been reported. The organs in which progress has been considered are vessels, where NOVA2 is located and works in a minor type of cells, the endothelial cells, which unexpectedly play major roles in angiogenesis. These considerations might be ultimately relevant for general progress in the field.

Future work could include various aspects of pathology in which NOVAs are of potential interest. At present, cancer [58,59,60,61,62,63,64,65,66,67,68,69,70,71,72,73,74,75] is already promising, whereas the state of brain diseases, such as neurodegeneration, epilepsy and psychiatric diseases [37,51,52,53,54,55,56,57], still appears preliminary. In the latter fields, interest concerns not only the progress of knowledge, but also the development of specific therapies based on both growing knowledge and innovative tools. Up to now, existing drugs have not been identified working on pathological NOVA processes, nor have new investments been made for the development of new therapies. In conclusion, NOVAs are interesting and the development of their potential is certainly promising.

## Figures and Tables

**Table 1:** Gene expression (TPM) of NOVA1 and NOVA 2 in human cells.

Tissues and Organs	NOVA1	NOVA2
Subcutaneous adipose	21	7
Brain		
amigdala	8	10
cingulate cortex	11	13
caudate	8	10
cerebellum	40	15
frontal cortex	20	19
hippocampus	9	10
hypothalamus	17	10
accumbens	9	13
putamen	7	9
spinal cord	13	6
substantia nigra	10	6
Breast mammary tissue	17	7
Cervix	11	5
Colon	9	2
Kidney	2	3
Lung	1	12
Pituitary	12	11
Thyroid	3	6
Uterus	9	6
Muscle	2	7

Data from Ref. [22,23]. TPM = transcripts per million. Reported values are those clearly appreciable in at least one NOVA.
